# Influence of proton pump inhibitors on the pathological response of rectal cancer: a multicentre study

**DOI:** 10.3332/ecancer.2023.1586

**Published:** 2023-08-10

**Authors:** Marcelle G Cesca, Erika Ruiz-Garcia, Rui Weschenfelder, Nathalia D’Agustini, Soledad Iseas, Romina Luca, Juan Manuel O’Connor, Renata D’Alpino, Allan A Pereira, Celso A Mello, Samuel Aguiar, Virgílio Souza e Silva, Rachel P Riechelmann

**Affiliations:** 1 A.C. Camargo Cancer Center, Antonio Prudente Street, 211, São Paulo, SP 10509001, Brazil; 2 Instituto Nacional de Cancerología, San Fernando Avenue, 22, Mexico City 14080, Mexico; 3 Hospital Moinhos de Vento, Ramiro Barcelos Street, 910, Porto Alegre, RS 90035-000, Brazil; 4 Hospital de Gastroenterología Dr. Carlos Bonorino Udaondo, Caseros Avenue, 2061, Buenos Aires C1264AAA CABA, Argentina; 5 Instituto Alexander Fleming, Crámer Street, 1180, Buenos Aires C1426ANZ, Argentina; 6 Hospital Alemão Oswaldo Cruz, Treze de Maio Street, 1815, São Paulo, SP 01323-020, Brazil; 7 Hospital Sírio Libanês Distrito Federal, SGAS 613 Street, Brasília 70200-730, Brazil

**Keywords:** capecitabine, 5-fluorouracil, rectal neoplasm, antacids, drug-drug interactions

## Abstract

**Background:**

The standard neoadjuvant therapy for rectal cancer involves fluoropyrimidines and radiotherapy and, most recently, total neoadjuvant therapy (TNT). A drug–drug interaction between fluoropyrimidines and proton-pump inhibitors (PPI) was suggested, with a negative impact on oncological outcomes in breast, colon and gastric cancers. Little is known about such an effect on rectal tumours. We aimed to evaluate the impact of PPI utilisation on the pathological response after chemoradiation for rectal cancer.

**Materials and methods:**

Retrospective multicentre study of rectal cancer patients treated with neoadjuvant chemoradiotherapy with capecitabine (cohort 1) or 5-fluororuracil (5-FU) (cohort 2); TNT with oxaliplatin-based regimens was allowed. The pathological response was considered a complete (ypCR) or complete + partial (ypCR + ypPR) according to American Joint Committee on Cancer. PPI use was considered at any time during the neoadjuvant period if concomitant to fluoropyrimidines.

**Results:**

From January 2007 to November 2020, 251 patients received capecitabine and 196 5-FU. The rates of PPI use in cohorts 1 and 2 were 20.3% and 26.5%, respectively. TNT was offered to 18.3% in cohort 1. PPI use did not influence ypCR in cohort 1 (yes versus no: 29.4% versus 19.5%; *p* = 0.13) or 2 (yes versus no: 25.0% versus 26.4%; *p* = 1.0). Similar ypCR + ypPR were observed in both cohorts 1 (76.5% versus 72.0%; *p* = 0.60) and 2 (86.5% versus 76.4%; *p* = 0.16). PPI use was not associated with pathological response in multivariable analysis. PPI users experienced more grade 3 or higher diarrhoea and infections.

**Conclusion:**

PPI concomitant to capecitabine/5-FU chemoradiation did not influence the pathological response in rectal cancer but was associated with more treatment-related adverse events.

## Introduction

Rectal cancer is the seventh most frequent malignant tumour worldwide, representing 3.8% of cancers diagnosed in 2020 and one-third of colorectal tumours [1, 2]. Neoadjuvant chemoradiotherapy with fluoropyrimidines has been the standard therapy for stage II and III rectal adenocarcinomas and, most recently, the total neoadjuvant chemotherapy (TNT) emerged as an additional alternative, with conflicting long-term efficacy results, depending on the chemotherapy and radiotherapy regimens used in the clinical trials [3–6]. Also, recent data demonstrated the feasibility of sparing tumours from radiotherapy if a decrease in size ≥20% after neoadjuvant chemotherapy is observed [7]. Capecitabine is an oral fluoropyrimidine, which demonstrated similar efficacy to 5-fluororuracil (5-FU) in colorectal cancers and is widely used in this scenario due to its posology [8, 9].

Oncological patients are more susceptible to drug-drug interactions (DDI) because of polypharmacy and pharmacokinetic alterations inherent to cancer and its directed therapies [10, 11]. While the number of oral antineoplastics has increased and studies on the impact of DDI on oncological outcomes have emerged, the clinical impact of DDI on oncological treatment is still underexplored [10]. There is a potential pharmacokinetic interaction between capecitabine and proton-pump inhibitors (PPI), in which the alkalinisation of gastric pH leads to a reduced absorption and dissolution of this drug [12, 13]. Although not proven *in vitro*, this combination demonstrated a negative impact on the overall and disease-free survival (DFS) in retrospective and prospective trials of patients with gastrointestinal tumours [12–15]. These results were not reproducible in all studies, so the existence of a DDI between PPI and fluoropyrimidines is controversial, which extends to 5-FU [16, 17].

Considering the standard use of capecitabine and 5-FU in neoadjuvant therapy for rectal cancer and the potential DDI between these chemotherapies and PPI, this study intended to evaluate whether the concomitant use of capecitabine or 5-FU and PPI influenced the pathological response after chemoradiation for rectal cancer.

## Material and methods

### Study design

This was a retrospective multicentre study of consecutive stage II–III rectal cancer patients treated with neoadjuvant fluoropyrimidine and radiotherapy (50–54 Gys) between January/2007 and November/2020 at one of the following centres: A.C. Camargo Cancer Center (Sao Paulo, Brazil), Hospital Moinhos de Vento (Porto Alegre, Brazil), Hospital Alemão Oswaldo Cruz (São Paulo, Brazil), Hospital Sírio Libanês (Distrito Federal, Brazil), Instituto Nacional de Cancerología (Ciudad de Mexico, Mexico), Hospital de Gastroenterología Dr. Carlos Bonorino Udaondo (Buenos Aires, Argentina) and Instituto Alexander Fleming (Buenos Aires, Argentina). Patients treated with 5-FU were exclusively from A.C. Camargo Cancer Center. The following data were collected from electronic medical records by investigators of each institution: demographic characteristics, comorbidities and medications intake (polypharmacy was defined as at least six medications per patient); tumour characteristics; neoadjuvant therapy details – chemotherapy and radiotherapy type and doses, dose reductions, treatment interruptions, grade ≥3 toxicities, and hospitalisations; PPI use during neoadjuvant period, type of PPI and time of use; pathological and clinical responses; adjuvant therapy; recurrences and death.

The study was carried out according to good clinical practice guidelines and Helsinki declaration and was approved by the ethics committees of each participating institution.

### Patient eligibility

Patients were eligible if they were at least 18 years old, had pathologically confirmed rectal adenocarcinoma, staged II–III, were treated with neoadjuvant fluoropyrimidine and radiotherapy followed by total mesorectal excision (TME). Cohort 1 included patients treated with neoadjuvant capecitabine and cohort 2, with 5-FU. TNT with capecitabine + oxaliplatin (CAPOX) and short course radiotherapy after TNT were allowed. Lack of information about PPI use was the exclusion criterion.

### Outcomes

The primary endpoint was pathological response: pathological complete response (ypCR), defined as pathological stage ypT0ypN0 by the American Joint Committee on Cancer (AJCC) 8th edition, and ypCR + pathological partial response (ypPR), which was defined by AJCC stage ypT0ypN0 or downstaging of the pathological ypT, ypN or stage in relation to clinical cT, cN or stage. The use of PPI was considered at any time and with any duration in the neoadjuvant period, and as an exploratory evaluation when a PPI was used for at least 50% of the neoadjuvant period (PPI >50; yes or no).

Secondary endpoints were clinical response defined by the assistant physician as complete [clinical complete response (cCR)], partial [clinical partial response (cPR)], stable [clinical stable disease (cSD)] or progression [clinical progressive disease (cPD)]; DFS, defined as the time between neoadjuvant therapy initiation and local or distant recurrence or death by any cause; toxicities, which were considered if any adverse event (AE) of grade 3 (G3) or higher by the common terminology criteria for adverse events version 5.0 or hospitalisation ≥24 hours occurred; and influence of PPI use on chemotherapy dose reductions, interruption or discontinuation.

### Statistical considerations

A predefined number of patients was not established, as all consecutive patients that fit the inclusion criteria and were treated during the predefined period, were recruited.

Descriptive statistics were used to define population characteristics and the primary outcomes. Comparisons of categorical variables were performed by *χ*^2^ bicaudal Pearson and Fisher’s Exact tests. Multivariable analyses of factors associated with ypCR or ypCR + ypPR were performed by binary logistic regression, adjusting for prognostic covariates (age, sex, Eastern Cooperative Oncology Group performance status, comorbidities, clinical stage, radiotherapy type and duration (5 weeks versus >5 weeks), interval from radiotherapy completion to TME (8 weeks versus >8 weeks), capecitabine dose intensity and clinical response). Covariates that resulted in *p* values <0.2 in univariable analyses were entered in the multivariable models; except for the clinical stage, which was forced in the adjusted analyses, irrespective of its *p*-value result in univariate analyses.

Continuous variables were described by means and medians and compared by *t* Student and Mann-Whitney U tests. Time-to-event variables were described by Kaplan-Meier curves and compared by the log-rank test. The median follow-up was calculated by the reverse Kaplan–Meier estimator.

95% confidence intervals (CI) were calculated for the relevant outcomes and bicaudal *p* values <0.05 were statistically significant. Analyses were performed by SPSS version 20.0 and were independent for cohorts 1 and 2.

## Results

Between 1st January 2007, and 30th November 2020, 575 patients were considered for inclusion and 447 were included: 251 in cohort 1 and 196 in cohort 2. The main reasons for patients’ exclusion were the adoption of the watch and wait strategy after neoadjuvant therapy and missing data (Appendix Figure 1a and b). The median follow-up was 22 months (95% CI: 19.1–24.9) in cohort 1 and 60 months (95%. CI: 65.2–63.8) in cohort 2. The median age was 58 and 59 years old for capecitabine and 5-FU cohorts, respectively, and polypharmacy was found in 9.6% and 9.7%. In cohort 1, 20.3% (*N* = 51) of patients were PPI users, and 12.7% used a PPI >50. In cohort 2, PPI users corresponded to 26.5% (*N* = 52) of patients and PPI >50 to 12.8% (Appendix Figure 2). Characteristics were well balanced between PPI users and non-users in cohort 2 and, in cohort 1, there were more female and older (≥65 years) patients in the PPI users’ group. Population characteristics are summarised in Table 1.

### Pathological response

ypCR and partial responses occurred in 21.5% and in 51.4% of patients in cohort 1 and in 26.0% and 53.1% of cohort 2. Both ypCR and ypCR + ypPR were similar for PPI and non-PPI users in both cohorts, as shown in Table 2. After excluding patients treated with TNT in cohort 1, the responses did not differ by PPI utilisation: PPI users and PPI non-users had 20.0% and 17.6% (*p* = 0.82) of ypCR, and 72.5% and 70.9% (*p* = 1.0) of ypCR + ypPR. Exploratory analysis of PPI >50 subgroups, also failed to demonstrate impact on the pathological response, both in cohort 1 (ypCR: 21.9% versus 21.5% (*p* = 1.0); ypCR + ypPR: 81.3% versus 71.7% (*p* = 0.29)) and in cohort 2 (ypCR: 25.9% versus 26.0 (*p* = 1.0); ypCR + ypPR: 81.5% versus 78.7% (*p* = 1.0)).

Clinical complete response and radiotherapy duration >5 weeks were independently associated with ypCR and ypCR + ypPR in cohort 1. In cohort 2, only clinical complete response was independently associated with improved ypCR; and female sex and absence of comorbidities, with ypCR + ypPR. PPI use was not associated with ypCR or ypCR + ypPR in any cohort (Table 3 and Supplementary Tables).

### Secondary outcomes

cCR and cPR were similar between PPI and non-PPI groups, both in cohort 1 (cCR: 30.4% versus 17.0% (*p* = 0.06); cCR + cPR: 84.8% versus 78.4% (*p* = 0.41)), and in cohort 2 (cCR: 18.8% versus 14.8% (*p* = 0.64); cCR + cPR: 79.2% versus 71.9% (*p* = 0.44)).

The estimated 3 year-DFS rate for PPI users in cohort 1 was 65.0% and for non-PPI, 69.7% (*p* = 0.96). In cohort 2, the 3 year-DFS rates for PPI and non-PPI groups were 72.7% and 64.0% (*p* = 0.44), respectively – Appendix Figure 3.

AE ≥ G3 occurred in 39.5% of patients and hospitalisations in 6.1% in cohort 1. These frequencies were 29.1% and 9.2% in cohort 2. AE ≥ G3 were predominant among PPI users in cohort 2 (40.4% versus 25.0%, *p* = 0.049), and, in both cohorts, diarrhoea ≥ G3 was more frequent among PPI users (cohort 1: 32.0% versus 18.7% (*p* = 0.05); cohort 2: 23.1% versus 9.0% (*p* = 0.01)). Rates of infections were also more common among PPI users in cohort 1 (8.0% versus 1.6% (*p* = 0.03)) and for non-PPI users in cohort 2 (1.9% versus 5.6% (*p* = 0.01)). There were no deaths related to AE. Table 4 summarises the AE. Capecitabine dose reductions were necessary for 27.9% patients and treatment interruption, or discontinuation occurred in 8.8%. 5-FU dose reductions and therapy interruption/discontinuation rates were 17.6% and 4.6%, respectively. PPI did not influence dose reductions or treatment discontinuation in both cohorts (Table 5).

## Discussion

This retrospective multicentre study observed that PPI utilisation was not associated with pathological response rates for rectal cancer patients treated with neoadjuvant fluoropyrimidines and radiotherapy, even after adjusting for potential confounders. Diarrhoea and infections were more frequent among PPI users.

The frequency of PPI intake in this study (20.3% for the capecitabine cohort and 26.5% for the 5-FU cohort) is in line with the literature, which describes gastric acid inhibitors use from 23.5% to 36.9% in colorectal cancer patients [18, 19]

The studies that evaluated potential DDI between PPI and capecitabine/5-FU in gastrointestinal tumours presented conflicting results [14, 20–28]. Chu *et al* [13] reported the detrimental effect of PPI in progression-free and overall survival in patients treated with CAPOX, but not CAPOX + lapatinib for metastatic gastric cancer. The rates of prior gastrectomy, which would make PPI ineffective, were not reported; and there are no explanations for lapatinib to overcome the DDI. Additionally, it was hypothesised that PPI could alter platins distribution and excretion through organic cation transporters inhibition, so the DDI reported could be in reality with oxaliplatin [29]. Wong *et al* [30] described inferior 3 year-DFS rate for PPI users during adjuvant CAPOX, but not FOLFOX, for stage III colorectal cancer patients. The study analysed capecitabine dose-intensity for PPI and non-PPI subgroups, but did not analyse other important potential confounders. In a *post hoc* analysis of six randomised clinical trials of metastatic colorectal cancer patients, PPI use was associated with poorer overall survival, which was maintained in the subanalysis of 5-FU group, but not in the capecitabine group [14].

Specifically in rectal cancer, three retrospective studies evaluated the effects of PPI and fluoropyrimidines during neoadjuvant chemoradiotherapy. Menon *et al* [21] did not find differences in pathological response or survival in relation to PPI use, both in capecitabine and 5-FU cohorts. Similar findings were observed in a retrospective French cohort of patients treated with neoadjuvant capecitabine + radiotherapy [27]. Zhang *et al* [16] described a trend of better responses to chemoradiotherapy (55.2% versus 36.5%; *p* = 0.072), and an improved 3 year-DFS in patients who used at least 200 mg of omeprazole during CAPOX concomitant to radiotherapy. This analysis was not pre-planned and for the entire cohort of PPI users, the results were in parallel to others (including ours), with no DDI been suggested [16].

The reasons for the above conflicting findings are unknown. We think that diverse chemotherapy regimens and populations, variable duration of PPI utilisation and doses may explain these heterogeneous results. Additionally, PPI may have their own detrimental effects in human health. For example, PPI use has been associated with inferior survival in colorectal cancer patients, possibly related to changes in fecal microbiota, increase in gastrin secretion with colonic polyp progression, and hypothetical endothelial function alterations [31–36]. PPI were also associated with increased incidence of diarrhoea and gastrointestinal infections due to intestinal microbiota modifications [34], what could explain the higher rates of diarrhoea linked to PPI use in our study.

Overall, we think the use of PPI during chemoradiation for rectal cancer should not be prohibited and likely does not influence oncological outcomes. This is because pharmacokinetic interaction between PPI and capecitabine could not be proven *in vitro* or *in vivo*, reinforcing our findings [12, 28, 37]. In addition, even the most potent PPI are incapable of alkalinising gastric pH to levels compatible with capecitabine ionisation [12]. A potential inhibition of gastrointestinal tumour cell proliferation by PPI through blockade of tumours vacuolar-H^+^ ATPase was demonstrated, as well as a potential sensibilisation of tumour cells to 5-FU when pre-treated (but not concomitantly treated) with PPI [38, 39]. These findings imply, but do not define, a DDI between 5-FU and PPI and highlights the need for *in vivo* research.

This study has limitations and is a hypothesis generator because of its retrospective design. Owing to the non-maleficence precept (due to potential detriment to survival shown before), this design seemed to be the most adequate. The study was subject to information biases, which were minimised by searches in medical, nurse, pharmaceutical and prescription records. Missing data precluded evaluation of PPI and capecitabine dose-intensity and therapy adhesion, the definition of chronic or sporadic use of PPI, and clinical response standardisation by images. Temporary biases must also be mentioned, considering that PPI use concomitantly to capecitabine probably reduced in the later years, after the publication of studies suggesting a potential DDI. Nevertheless, we decided to focus on pathological response to have a more accurate evaluation of the potential effect of PPI on tumour response and avoid subjective interpretation of watchful wait approaches.

Future studies focusing on the impact of DDI on oncological outcomes and healthcare costs are needed, especially due to the wide availability of oral antineoplastics. To define the occurrence of DDI more precisely, the inclusion of pharmacokinetic analysis is crucial.

## Conclusion

In conclusion, in this multicentre study conducted in Latin America, PPI utilisation concomitant to capecitabine or 5-FU did not influence pathological response in these cohorts of rectal cancer patients. We think that for those with concomitant pathologies that require PPI intake, the use of PPI appears to be safe.

## Author contributions

Marcelle G Cesca and Rachel P Riechelmann contributed as lead writers of the paper. The remaining authors contributed equally to manuscript writing and final approval. All authors contributed to data collection and/or interpretation.

## Conflicts of interest

No conflicts of interest to declare.

## Funding

This research did not receive any specific grant from funding agencies in the public, commercial, or not-for-profit sectors.

## Figures and Tables

**Figure 1.**
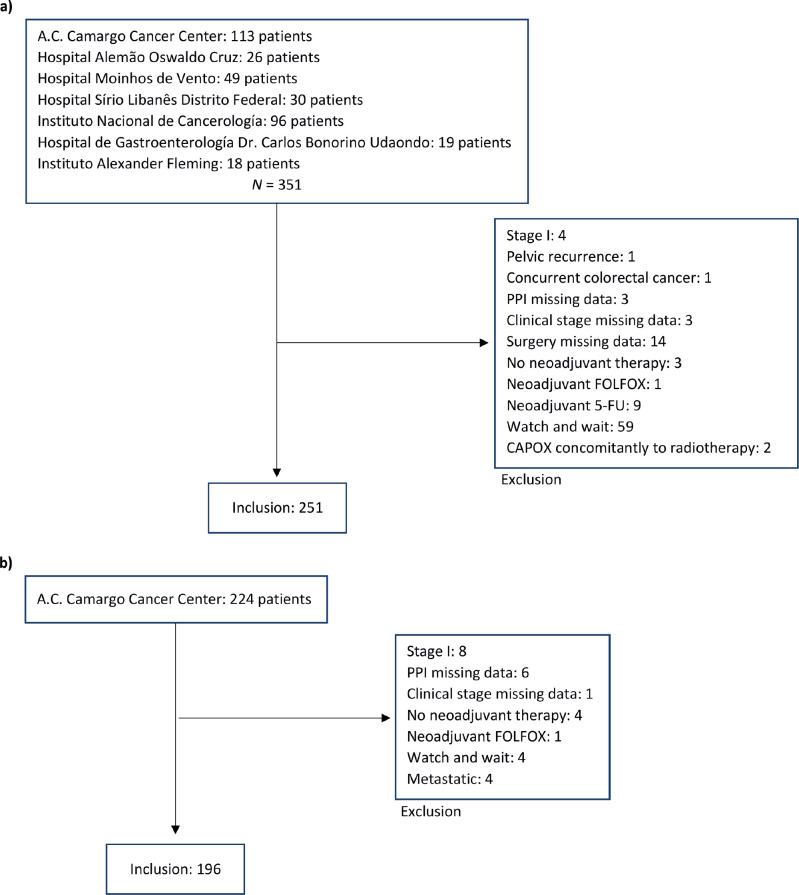
Flow diagram with included and excluded patients. (a): Inclusion and exclusion in cohort 1, capecitabine; (b): Inclusion and exclusion in cohort 2, 5-FU.

**Figure 2.**
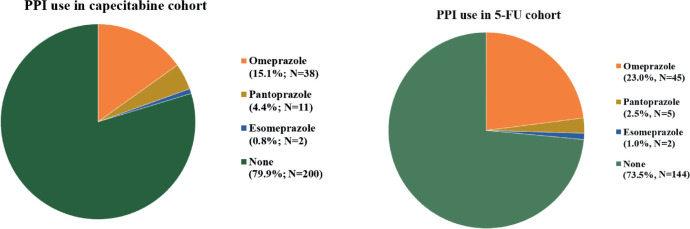
PPI use.

**Figure 3.**
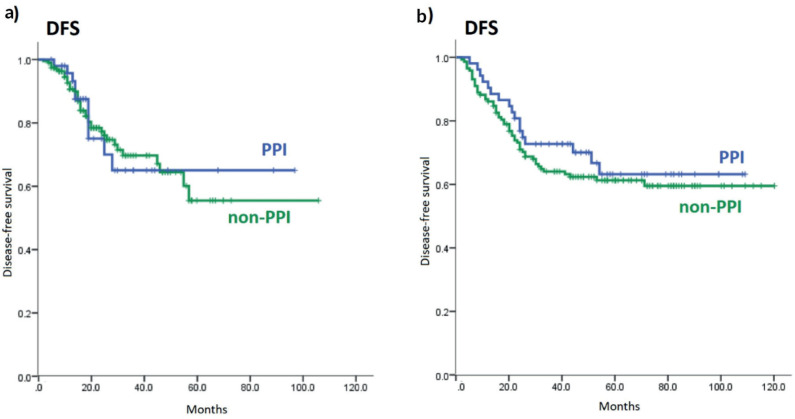
DFS of PPI and non-PPI subgroups. (a): DFS of PPI and non-PPI subgroups in cohort 1; (b): DFS of PPI and non-PPI subgroups in cohort 2. DFS: Disease-free survival; PPI: proton-pump inhibitors.

**Supplementary Table 1.** Univariable and multivariable analysis of cohort 1.

	ypCR					ypCR + ypPR				
	Univariable analysis			Multivariable analysis		Univariable analysis			Multivariable analysis	
	ypCR (54)	Non-ypCR (197)	*p* value	OR (95%CI)	*p* value	ypCR + ypPR (183)	Non-ypCR + ypPR (68)	*p* value	OR (95%CI)	*p* value
Age < 65y ≥ 65y Missing	31 (57.4%) 23 (42.6%) 0	136 (69.4%) 60 (30.6%) 0	0.10	0.51 (0.22–1.17) 1	0.11	123 (67.6%) 59 (32.4%) 1	44 (64.7%) 24 (35.3%) 0	0.76	-	-
Sex Female Male	25 (46.3%) 19 (53.7%)	78 (39.6%) 119 (60.4%)	0.43	-	-	80 (43.7%) 103 (56.3%)	23 (33.8%) 45 (66.2%)	0.19	1.39 (0.70–2.79) 1	0.35
ECOG 0 ≥ 1 Missing	18 (33.3%) 36 (66.7%) 0	65 (33.7%) 128 (66.3%) 3	1.00	-	-	60 (33.5%) 119 (66.5%) 4	23 (33.8%) 45 (66.2%) 0	1.00	-	-
Comorbidities No Yes	22 (40.7%) 32 (59.3%)	80 (40.6%) 117 (59.4%)	1.00	-	-	79 (43.2%) 104 (56.8%)	23 (33.8%) 45 (66.2%)	0.19	1.60 (0.78–3.29) 1	0.20
Clinical stage II III	11 (20.4%) 43 (79.6%)	39 (19.8%) 158 (80.2%)	1.00	0.76 (0.29–2.03) 1	0.59	28 (15.3%) 155 (84.7%)	22 (32.4%) 46 (67.6%)	**0.004**	0.49 (0.22–1.09) 1	0.08
PPI No Yes	39 (72.1%) 15 (27.8%)	161 (81.7%) 36 (18.3%)	0.13	1 1.101 (0.42–2.91)	0.85	144 (78.7%) 39 (21.3%)	56 (82.4%) 12 (17.6%)	0.60	-	-
PPI >50 No Yes	47 (87.0%) 7 (13.0%)	172 (87.3%) 25 (12.7%)	1.00	-	-	157 (85.8%) 26 (14.2%)	62 (91.2%) 6 (8.8%)	0.29	-	-
Radiotherap**y** 3D Other Missing	42 (77.8%) 12 (22.2%) 0	143 (76.5%) 44 (23.5%) 9	1.00	-	-	143 (80.3%) 35 (19.7%) 5	42 (66.7%) 21 (33.3%) 5	**0.04**	1 0.39 (0.18–0.83)	**0.01**
Time RT – surgery ≤ 8 weeks > 8 weeks Missing	7 (13.2%) 46 (86.8%) 1	21 (11.1%) 168 (88.9%) 7	0.63	-	-	20 (11.1%) 160 (88.9%) 3	8 (12.9%) 54 (87.1%) 6	0.65	0.52 (0.25–1.10) 1	0.08
RT duration ≤ 5 weeks > 5 weeks Missing	39 (72.2%) 15 (27.8%) 0	119 (61.3%) 75 (38.7%) 3	0.15	2.60 (1.06–6.38) 1	**0.04**	109 (60.2%) 72 (39.8%) 2	49 (73.1%) 18 (26.9%) 1	0.07	-	-
Dose-intensity cap 100% < 100% Missing	37 (71.2%) 15 (28.8%) 2	133 (70.7%) 55 (29.3%) 8	1.00	-	-	124 (70.1%) 53 (29.9%) 6	46 (73.0%) 17 (27.0%) 5	0.75	-	-
Clinical response^a^ No Yes Missing	20 (40.0%) 30 (60.0%) 4	154 (92.2%) 13 (7.8%) 30	**<0.001**	1 21.67 (8.92–52.68)	**<0.001**	22 (13.8%) 137 (86.2%) 24	22 (37.9%) 36 (62.1%) 10	**<0.001**	1 4.09 (1.91–8.74)	**<0.001**

a Clinical complete response was used for ypCR analysis and clinical complete + partial response for ypCR + ypPR analysis

ypCR: pathological complete response; ypPR: pathological partial response; OR: odds ratio; 95%CI: 95% CI; y: years; PPI: proton-pump inhibitors; PPI > 50: use of proton-pump inhibitors > 50% of neoadjuvant therapy duration; RT: radiotherapy; Cap: capecitabine

The bold values represent statistical significant factors associated with ypCR and ypCR+ypPR in univariable and multivariable analysis in cohort 1

**Supplementary Table 2.** Univariable and multivariable analysis of cohort 2.

	ypCR					ypCR + ypPR				
	Univariable analysis			Multivariable analysis		Univariable analysis			Multivariable analysis	
	ypCR (51)	Non-ypCR (145)	*p* value	OR (95%CI)	*p* value	ypCR + ypPR (155)	Non-ypCR + ypPR (41)	*p* value	OR (95%CI)	*p* value
Age < 65y ≥ 65y	36 (70.6%) 15 (29.4%)	97 (66.9%) 48 (33.1%)	0.73	-	-	106 (68.4%) 49 (31.6%)	27 (65.9%) 14 (34.1%)	0.85	-	-
Sex Female Male	23 (45.1%) 28 (54.9%)	57 (39.3%) 88 (60.7%)	0.51	-	-	69 (44.5%) 86 (55.5%)	11 (26.8%) 30 (73.2%)	0.049	2.22 (1.05–4.85) 1	0.046
ECOG 0 ≥ 1 Missing	35 (70.0%) 15 (30.0%) 1	96 (66.2%) 49 (33.8%) 0	0.73	-	-	102 (66.2%) 52 (33.8%) 1	29 (70.7%) 12 (29.3%) 0	0.71	-	-
Comorbidities No Yes	10 (19.6%) 41 (80.4%)	43 (29.7%) 102 (70.3%)	0.20	-	-	47 (30.3%) 108 (69.7%)	6 (14.6%) 35 (85.4%)	0.049	1 2.62 (1.01–6.79)	0.047
Clinical stage II III	12 (23.5%) 39 (76.5%)	32 (22.1%) 113 (77.9%)	0.85	1.30 (0.55–3.07) 1	0.54	31 (20.0%) 124 (80.0%)	13 (31.7%) 28 (68.3%)	0.14	0.50 (0.22–1.05) 1	0.09
PPI No Yes	38 (74.5%) 13 (25.5%)	106 (73.1%) 39 (26.9%)	1.00	-	-	110 (71.0%) 45 (29.0%)	34 (82.9%) 7 (17.1%)	0.16	0.48 (0.19–1.20) 1	0.12
PPI >50 No Yes	44 (86.3%) 7 (13.7%)	125 (86.2%) 20 (13.8%)	1.00	-	-	133 (85.8%) 22 (14.2%)	36 (87.8%) 5 (12.2%)	1.00	-	-
Radiotherapy 3D Other	2 (3.9%) 49 (96.1%)	18 (12.4%) 127 (87.6%)	0.11	1 0.32 (0.07-1.51)	0.15	16 (10.3%) 139 (89.7%)	4 (9.8%) 37 (90.2%)	1.00	-	-
Time RT – surgery ≤ 8 weeks > 8 weeks	7 (13.7%) 44 (86.3%)	21 (14.5%) 124 (85.5%)	1.00	-	-	25 (16.1%) 130 (83.9%)	3 (7.3%) 38 (92.7%)	0.21	-	-
RT duration ≤ 5 weeks > 5 weeks	21 (41.2%) 30 (58.8%)	66 (45.5%) 79 (54.5%)	0.63	-	-	69 (44.5%) 86 (55.5%)	18 (43.9%) 23 (56.1%)	1.00	-	-
Dose-intensity 5-FU 100% < 100%	41 (80.4%) 10 (19.6%)	121 (83.4%) 24 (16.6%)	0.67	-	-	126 (81.3%) 29 (18.7%)	36 (87.8%) 5 (12.2%)	0.49	-	-
Infusional 5-FU No Yes	7 (13.7%) 44 (86.3%)	26 (17.9%) 119 (82.1%)	0.66	-	-	27 (11.4%) 128 (82.6%)	6 (14.6%) 35 (85.4%)	0.82	-	-
Clinical response^a^ No Yes Missing	30 (65.2%) 16 (34.8%) 5	118 (90.8%) 12 (9.2%) 15	<0.001	1 15.21 (2.21-12.31)	<0.001	38 (27.1%) 102 (72.9%) 15	8 (22.2%) 28 (77.8%) 5	0.67	-	-

a Clinical complete response was used for ypCR analysis and clinical complete + partial response for ypCR + ypPR analysis

ypCR: pathological complete response; ypPR: pathological partial response; OR: odds ratio; 95%CI: 95% CI; y: years; PPI: proton-pump inhibitors; PPI > 50: use of proton-pump inhibitors > 50% of neoadjuvant therapy duration; RT: radiotherapy; 5-FU: 5-fluorouracil

The bold values in represent statistical significant factors associated with ypCR and ypCR+ypPR in univariable and multivariable analysis in cohort 2

**Supplementary Table 3.** AE ≥ grade 3 and hospitalisation considering PPI >50.

	Cohort 1: Capecitabine^a^				Cohort 2: 5-FU			
	Overall (243)	PPI >50 (31)	Non-PPI (212)	*p* value	Overall (196)	PPI >50 (27)	Non-PPI (169)	*p* value
All	39.5%	35.5%	40.1%	0.70	29.1%	40.7%	27.2%	0.17
Diarrhoea	21.4%	22.6%	21.2%	0.89	12.8%	18.5%	11.8%	**0.33**
HFS	16.0%	9.7%	17.0%	0.30	0	0	0	-
Infection	2.9%	3.2%	2.8%	1.00	4.6%	0	5.3%	0.22
Rectitis	6.6%	9.7%	6.1%	0.46	8.7%	11.1%	8.3%	0.63
Cystitis	18.1%	3.2%	20.3%	**0.02**	0.5%	0	0.6%	0.69
Oral mucositis	6.2%	3.2%	6.6%	0.46	9.7%	11.5%	9.0%	0.69
Other	2.5%	3.2%	2.4%	0.77	5.6%	11.1%	4.7%	0.18
Hospitalisation	6.1%	3.2%	6.5%	0.47	9.2%	11.1%	8.9%	0.71
ICU	1.2%	3.1%	0.9%	0.28	1.5%	3.7%	1.2%	0.32

a Missing data of 8 of 251 patients

PPI>50: use of proton-pump inhibitors for at least 50% of the neoadjuvant period; PPI: proton-pump inhibitors; HFS: hand and foot syndrome; ICU: Intensive care unit

The bold values represent statistical significant differences in the occurrence of AE>= grade 3 and hospitalization between PPI>50 and non-PPI>50 groups

**Table 1.** Population baseline and treatment characteristics.

	Cohort 1: capecitabine				Cohort 2: 5-FU			
	Overall (251), N(%)	PPI (51), N(%)	Non-PPI (200), N(%)	*p* value	Overall (196), N(%)	PPI (52), N(%)	Non-PPI (144), N(%)	*p* value
Age Median (range) ≥65 years Missing	58 (21–90) 83 (33.1) 1 (0.4)	64 (31–90) 25 (49.0) 0	57 (21–89) 58 (29.0) 1 (0.5)	0.05 **0.01**	59 (22–92) 63 (32.1) 0	59 (29–84) 17 (32.7) 0	60 (22–92) 46 (31.9) 0	0.96 1.00
Sex Female Male	103 (41.0) 148 (59.0)	30 (58.8) 21 (41.2)	73 (36.5) 127 (63.5)	**0.006**	80 (40.8) 116 (59.2)	26 (50.0) 26 (50.0)	54 (37.5) 90 (62.5)	0.14
ECOG 0 ≥ 1 Missing	83 (33.1) 164 (65.3) 4 (1.6)	11 (21.6) 39 (76.5) 1 (1.9)	72 (36.0) 125 (62.5) 3 (1.5)	0.65	131 (66.8) 64 (32.6) 1 (0.8)	31 (59.6) 21 (40.4) 0	100 (69.4) 43 (29.9) 1 (0.7)	0.23
Comorbidities	149 (59.4)	31 (60.8)	118 (59.0)	0.87	143 (73.0)	40 (76.9)	103 (71.5)	0.58
clinical stage II III	50 (19.9) 201 (80.1)	12 (23.5) 39 (76.5)	38 (19.0) 162 (81.0)	0.56	44 (22.4) 152 (77.6)	12 (23.1) 40 (76.9)	32 (22.2) 112 (77.8)	1.00
Localisation Distal Middle Upper Missing	134 (53.4) 99 (39.4) 15 (6.0) 3 (1.2)	31 (60.8) 17 (33.3) 3 (5.9) 0	103 (51.5) 82 (41.0) 12 (6.0) 3 (1.5)	0.54	113 (57.7) 74 (37.7) 9 (4.6) 0	29 (55.8) 20 (38.5) 3 (4.6) 0	84 (58.3) 54 (37.5) 6 (4.2) 0	0.87
Differentiation Well/moderate Poor Missing	206 (82.1) 35 (13.9) 10 (4.0)	44 (86.3) 5 (9.8) 2 (3.9)	162 (81.0) 30 (15.0) 8 (4.0)	0.49	175 (89.3) 12 (6.1) 9 (4.6)	42 (80.8) 6 (11.5) 4 (7.7)	133 (92.3) 6 (4.2) 5 (3.5)	0.08
CAPOX TNT	46 (18.3)	2 (5.3)	35 (17.5)	0.54	-	-	-	-
radiotherapy 2D 3D IMRT Short course Missing	1 (0.3) 185 (73.7) 30 (12.0) 25 (10.0) 10 (4.0)	0 34 (66.6) 6 (11.8) 9 (17.6) 2 (4.0)	1 (0.5) 151 (75.5) 24 (12.0) 16 (8.0) 8 (4.0)	0.21	5 (2.5) 176 (89.8) 15 (7.7) 0 0	1 (1.9) 50 (96.2) 1 (1.9) 0 0	4 (2.8) 126 (87.5) 14 (9.7) 0 0	0.18
ΔT Neo-TME (median, weeks) Neo ending RT ending	13 (11.4–14.6) 14 (12.4–15.6)	13 (9.5–16.5) 16 (14.3–17.7)	12 (10.9–13.1) 13 (11.6–14.4)	0.35 0.85	- 10 (9.6–10.4)	- 11 (9.7–12.3)	- 10 (9.6–10.4)	0.10
Adjuvant therapy None Capecitabine CAPOX 5-FU FOLFOX Missing	85 (33.9) 62 (24.7) 64 (25.5) 0 38 (15.1) 2 (0.8)	18 (35.3) 14 (27.5) 15 (29.4) 0 4 (7.8) 0	67 (33.5) 48 (24.0) 49 (24.5) 0 34 (17.0) 2 (1.0)	0.42	54 (27.6) 10 (5.1) 12 (6.1) 34 (17.3) 86 (43.9) 0	16 (30.8) 4 (7.7) 2 (3.8) 12 (23.1) 18 (34.6) 0	38 (26.4) 6 (4.2) 10 (6.9) 22 (15.3) 68 (47.2) 0	0.34

PPI: proton-pump inhibitors; y: years; CAPOX: capecitabine + oxaliplatin; TNT: total neoadjuvant therapy; IMRT: intensity modulated radiotherapy; ΔT: median time; Neo: neoadjuvant therapy ending; RT: radiotherapy: TME: total mesorectal excision; FOLFOX: 5-Fluorouracil + oxaliplatin

The bold values represent statistical significant differences in baseline characteristics between PPI and non-PPI groups

**Table 2.** Pathological response.

	Cohort 1: Capecitabine			Cohort 2: 5-FU		
	PPI (51)	Non-PPI (200)	*p* value	PPI (52)	Non-PPI (144)	*p* value
ypCR	29.4%	19.5%	0.13	25.0%	26.4%	1.00
ypPR	47.1%	52.5%		61.5%	50.0%	
ypCR + ypPR	76.5%	72.0%	0.60	86.5%	76.4%	0.16
ypSD	17.6%	18.5%		7.7%	13.2%	
ypPD	8.8%	9.5%		5.8%	10.4%	

PPI: proton-pump inhibitors; ypCR: pathological complete response; ypPR: pathological partial response; ypSD: pathological stable disease; ypPD: pathological progressive disease

**Table 3.** Multivariable analysis of cohorts 1 and 2.

	Cohort 1: Capecitabine				Cohort 2: 5-FU			
	ypCR		ypCR + ypPR		ypCR		ypCR + ypPR	
	OR (95%CI)	*p* value	OR (95%CI)	*p* value	OR (95%CI)	*p* value	OR (95%CI)	*p* value
Age (≥ 65 years)	0.51 (0.22–1.17)	0.11	-	-	-	-	-	-
Sex (male)	-	-	1.39 (0.70–2.79)	0.35	-	-	2.22 (1.05–4.85)	**0.046**
Comorbidities (yes)	-	-	1.60 (0.78–3.29)	0.20	-	-	2.62 (1.01–6.79)	**0.047**
Clinical stage (III)	0.76 (0.29–2.03)	0.59	0.49 (0.22–1.09)	0.08	1.30 (0.55–3.07)	0.54	0.50 (0.22–1.05)	0.09
PPI (no)	1.10 (0.42–2.91)	0.85	-	-	-	-	2.08 (0.83–5.20)	0.12
Radiotherapy (3D)	-	-	0.39 (0.18–0.83)	**0.01**	0.32 (0.07–1.51)	0.15	-	-
Time RT – Surgery (> 8 weeks)	-	-	0.52 (0.25–1.10)	0.08	-	-	-	-
RT duration (>5 weeks)	2.60 (1.06–6.38)	**0.04**	-	-	-	-	-	-
Clinical response (yes)^a^	21.67 (8.92–52.68)	**<0.001**	4.09 (1.91–8.74)	<0.001	15.21 (2.21–12.31)	**<0.001**	-	-

a Clinical complete response was used for ypCR analysis and clinical complete + partial response for ypCR + ypPR analysis

OR: odds ratio; 95% CI: 95% CI; y: years; PPI: proton-pump inhibitors; RT: radiotherapy

The bold values represent statistical significant factors associated with ypCR and ypCR+ypPR in multivariable analysis in both cohorts 1 and 2

**Table 4.** AE ≥ grade 3 and hospitalisation.

	Cohort 1: Capecitabine^a^				Cohort 2: 5-FU			
	Overall (243)	PPI (50)	Non-PPI (193)	*p* value	Overall (196)	PPI (52)	Non-PPI (144)	*p* value
All	39.5%	48.0%	37.3%	0.19	29.1%	40.4%	25.0%	**0.049**
Diarrhoea	21.4%	32.0%	18.7%	0.05	12.8%	23.1%	9.0%	**0.01**
HFS	16.0%	22.0%	14.5%	0.20	0	0	0	-
Infection	2.9%	8.0%	1.6%	**0.03**	4.6%	1.9%	5.6%	**0.01**
Rectitis	6.6%	6.0%	6.7%	1.00	8.7%	11.5%	7.6%	0.71
Cystitis	18.1%	24.0%	16.6%	0.22	0.5%	0	0.7%	0.34
Oral mucositis	6.2%	8.0%	5.7%	0.52	9.7%	11.5%	9.0%	0.69
Other	2.5%	2.0%	2.6%	1.00	5.6%	7.7%	4.9%	1.00
Hospitalisation Days (median)	6.1% 2 (1–22)	8.0% 1.5 (1–22)	5.6% 2 (1–16)	0.51 1.00	9.2% 7 (2–25)	13.5% 7 (2–12)	7.6% 7 (3–25)	0.26 0.48
ICU	1.2%	2.0%	1.0%	0.50	1.5%	3.8%	0.7%	0.17

a Missing data of 8 of 251 patients

PPI: proton-pump inhibitors; HFS: hand and foot syndrome; ICU: Intensive care unit

The bold values represent statistical significant differences in the occurrence of AE>= grade 3 and hospitalization between PPI and non-PPI groups

**Table 5.** Dose reductions and treatment interruption or discontinuation.

	Cohort 1: Capecitabine				Cohort 2: 5-FU			
	Overall (251)	PPI (51)	Non-PPI (200)	*p* value	Overall (196)	PPI (52)	Non-PPI (144)	*p* value
Dose reduction No Yes Missing	170 (67.7%) 70 (27.9%) 11 (4.4%)	36 (73.5%) 13 (26.5%) 2	134 (70.2%) 57 (29.8%) 9	0.73	162 (82.7%) 34 (17.6%) 0	43 (82.7%) 9 (17.3%) 0	119 (82.6%) 25 (17.4%) 0	1.00
Pause for toxicity^a^ No Yes Missing	229 (91.2%) 22 (8.8%) 9 (3.6%)	46 (93.9%) 3 (6.1%) 2	173 (89.6%) 20 (10.4%) 7	0.58	187 (95.4%) 9 (4.6%) 0	51 (98.1%) 1 (1.9%) 0	136 (94.4%) 8 (5.6%) 0	0.45

a Temporary pause or definitive discontinuation

PPI: proton-pump inhibitors
